# Weighing In

**DOI:** 10.5811/westjem.18690

**Published:** 2024-10-04

**Authors:** Iyesatta M. Emeli, Patrick G. Meloy

**Affiliations:** Emory University School of Medicine, Department of Emergency Medicine, Atlanta, Georgia

“What is your patient’s biggest problem?”

This simple question was posed two decades ago by an attending physician to a medical student rotating in a family medicine clinic. The patient had the common trio of diabetes, hypertension, and hyperlipidemia, and the student went through this list of conditions. The student’s attending, however, remained unconvinced. “Obesity,” the attending finally said. “Obesity is this patient’s biggest problem.” The patient was overweight, yes, but in that comfortable way that we overlook in our family members, in our friends, and in ourselves. That was the first time in that medical student’s training that any clinician had put weight on par with, and indeed above, other established chronic medical conditions. It was also the last time.

This vignette highlights our lack of routine acknowledgment of obesity as a disease.

Obesity is a disease,^1^ ^,^ ^2^ just as diabetes, hypertension, and hyperlipidemia are diseases. Obesity is also a public health crisis, both locally and globally.^3^ In the United States, up to one in three adults and one in six children are affected, and the incidence and prevalence has been increasing steadily over the past 20 years.^3^ The increase in morbidity and mortality due to obesity cannot be understated: obese adults have lower life expectancy, lower quality of life, and substantial increases in healthcare costs.^3^ Additionally, obesity increases the risk of being diagnosed with other lifelong diseases such as diabetes, hypertension, coronary artery disease, myocardial infarction, and stroke.^4^ In addition, the combination of obesity and other comorbid conditions, eg, smoking, can increase the risk of death by up to 11 times that of a non-obese, non-smoker.^5^ It is through these lenses that public health officials have recognized obesity as an alarming disease process that threatens the lives of both young and old and is increasingly prevalent in US healthcare.

As emergency physicians, we do not, for the most part, have long-term relationships with our patients. We expect primary care physicians and those who provide ongoing care to make it their business to counsel on weight loss. Unfortunately, the data says otherwise. According to the US Centers for Disease Control and Prevention (CDC), even in physician visits made specifically for obesity, weight-related health education was only offered half of the time.^6^

Emergency departments (ED) are the safety nets of the US healthcare system, caring for acute, unscheduled patients regardless of ability to pay. While many ED visits are subsequently deemed to be non-emergent, they still require a timely evaluation and involve some, albeit not as comprehensive, preventative care interventions. We tell our patients to quit smoking, practice safe sex, take seizure precautions, wear their seat belts and bicycle helmets, and get their COVID-19 shots. Charts are littered with “dot phrases” such as the following: “Patient is counseled for tobacco cessation”; “Will admit for TIA rule-out and initiate statin therapy”; “Patient is treated empirically for urethritis and is counseled on safe sexual practices”; or “Patient is given information on low-salt diet for congestive heart failure.” However, advice on proper diet and exercise is uncommon; in our clinical experience, we tend to address obesity only when it is morbid and when it disrupts our intended care plan; for example, when the patient is too overweight for a stretcher, a scanner, or a procedure. Should we be talking about weight in the ED? If the answer is yes, then what is the best way to have such discussions?

The question of the medical community’s approach to obesity is important for two reasons. The first is what the COVID-19 pandemic taught us about obesity. The COVID-19 pandemic brought many of the failures in US healthcare into sharp focus, and the obesity epidemic was one of them. Our collective personal experience of clinical medicine during the pandemic made it clear: COVID-19 and obesity make catastrophic bedfellows. Of the 2.5 million COVID-19 deaths reported by February 2021, 2.2 million occurred in countries where more than half the population had a body mass index over 25.^7^ Additionally, the CDC reported that obesity tripled the rate of hospitalization for COVID-19.^8^ The second reason this topic is timely is because of the increasing move to normalize obesity,^9^ a move that makes it less likely for physicians and patients alike to recognize obesity for what it is: a disease. This type of normalization threatens to make our opening vignette the continued standard.

Of course, the issue of obesity is complex. Obesity’s causes are multifactorial, and any treatment must, as such, be multi-leveled.^10^ In preventative health, the social ecology model (SEM) —a framework for health promotion that uses a multitiered approach to address the interplay between multiple factors that influence a given problem—would be employed to tackle obesity at the population level. The SEM postulates that conditions such as obesity are shaped at several levels: the individual, interpersonal, organizational, community, and public policy levels.^11^ The SEM pivots away from an isolated attentiveness on individual behavior to a more encompassing understanding of tiers of influence. The SEM promotes interventions on the social determinants of health, the forces and systems that shape the well-being of a population. This means not only supporting individual efforts toward healthy diet and regular physical activity but also changing the context in which behaviors arise and are sustained. Thus, public health officials can include trans fat elimination in processed foods, community infrastructure that promotes greater physical activity, improving access to healthy foods, etc, as potential interventions.

Given all this, one might decide that obesity is too complex to deal with in the ED. No patient to our knowledge has ever signed into an ED with a chief complaint of “I am overweight”; that is to say, a patient’s weight is never the reason they choose to come to the ED. So, why try to discuss what many consider a sensitive matter and risk jeopardizing one’s rapport with a patient? We believe the ED, while certainly not the end-all-be-all, is still an important tier of influence; so, such discussions do have a place there.

How then can we talk to our patients about obesity in the ED? There are guidelines and practical approaches published in the medical literature that provide a framework in the primary care setting for engaging with patients about their weight.^12^ ^,^ ^13^ The Canadian Obesity Network developed the “5As” of obesity counseling; ask, assess, advise, agree and assist.^14^ Given the brevity of the ED encounter, we suggest the ED approach should be an abbreviated version of the 5As, perhaps better termed the 3As. It would involve first **asking** permission to discuss the patient’s health risk, one risk being that they are well above ideal body weight. Sensitivity and non-judgmental language are paramount. If the patient is open to a conversation, one could go on to **advise** them on how their obesity can hurt them. Finally, it would be important to **assist** the patient by providing resources for outpatient follow-up and management. This is not unlike what we already do when we provide brief counseling to patients for other health concerns. The difference is that other health concerns are recognized by both patients and clinicians as addressable issues, while there is a pervasive lack of recognition of obesity as a disease.

But obesity is a disease. It behooves us now, more than ever, to address it as such.
